# Benefits of Krill Oil Supplementation During Alternate‐Day Fasting in Adults With Overweight and Obesity: A Randomized Trial

**DOI:** 10.1002/oby.24354

**Published:** 2025-07-16

**Authors:** Mansour Alblaji, Stuart R. Gray, Taibah Almesbehi, Douglas J. Morrison, Dalia Malkova

**Affiliations:** ^1^ Human Nutrition, School of Medicine, Dentistry and Nursing, College of Medical, Veterinary, and Life Sciences University of Glasgow Glasgow UK; ^2^ Department of Basic Health Sciences, College of Applied Medical Sciences Qassim University Buraydah Saudi Arabia; ^3^ School of Cardiovascular and Metabolic Health, College of Medical, Veterinary and Life Sciences University of Glasgow Glasgow UK; ^4^ Institute of Sports Science and Innovation Lithuanian Sports University Kaunas Lithuania; ^5^ Scottish Universities Environmental Research Centre (SUERC) University of Glasgow East Kilbride UK

**Keywords:** body composition, caloric restriction, long‐chain n‐3 polyunsaturated fatty acids, muscle function, weight loss

## Abstract

**Objective:**

To investigate the effect of krill oil (KO) supplementation during alternate‐day fasting (ADF) on body composition and muscle function in healthy adults with overweight.

**Methods:**

In a randomized trial, during the 8‐week ADF, participants consumed four capsules per day containing krill oil (KO group) or vegetable oil (placebo group). Each capsule of KO contained 191 mg EPA, 94 mg DHA, 78 mg choline, and 100 mcg astaxanthin. Body mass, fat‐free mass (FFM), and handgrip strength (HGS) were measured before and after the intervention. Data were analyzed using ANOVA.

**Results:**

The study was completed by 41 (25 women and 16 men) participants (age: 39 ± 10 years, BMI: 31.1 ± 4.2 kg/m^2^). Body weight reduction was not different (*p* > 0.05) between groups (KO, −4.6 ± 1.4 kg; Placebo, −4.5 ± 1.9 kg). The KO group had no change (*p* > 0.05) in FFM (−0.2 ± 0.9 kg) or HGS (−0.2 ± 0.5 kg). The placebo group experienced a reduction (*p* < 0.05) in FFM (−1.2 ± 2.0 kg) and HGS (−0.9 ± 0.7 kg). Changes in FFM and HGS were different (*p* < 0.05) between groups.

**Conclusions:**

KO supplementation during body weight loss attenuates the decline in FFM and muscle strength.

**Trial Registration:** ClinicalTrials.gov Identifier (NCT06001632)

**Summary**

- Diet‐induced weight loss reduces fat‐free mass (FFM) and muscle strength, which may negatively impact metabolic health and physical function. Supplementation with long‐chain n3 polyunsaturated fatty acids (LCn‐3 PUFA) improves muscle mass and function, but studies during weight loss are limited.
- The current study demonstrated that supplementation with krill oil, as a source of LCn‐3 PUFA, during diet‐induced weight loss attenuated the reduction in FFM and muscle strength, improved functional performance, and reduced inflammatory markers.
- Krill oil supplementation may offer a beneficial strategy to enhance the quality of weight loss by minimizing FFM and muscle strength loss. These findings support the implementation of krill oil supplementation during weight loss in adults living with overweight and obesity.

## 1 Introduction

Obesity, a major global public health issue, is characterized by excessive fat accumulation and is linked to various adverse health outcomes, including type 2 diabetes, hypertension, cardiovascular disease, and musculoskeletal issues [1]. One of the solutions to the pandemic of overweight and obesity is diet‐induced weight loss, with a systematic review and meta‐analysis highlighting that these interventions result in significant weight loss of on average around 4–5 kg [2]. This level of weight loss results in improvements in cardiovascular risk factors [2].

However, diet‐induced weight loss leads to reductions in both fat mass and fat‐free mass (FFM), with about 25%–30% of body mass loss attributed to the decrease in FFM [3, 4]. This decline in FFM is primarily associated with reduced muscle mass, potentially leading to diminished muscle strength [5]. As muscle also has an important metabolic role, this reduction in FFM and muscle mass could potentially have adverse effects on metabolic health [6, 7]. Therefore, although individuals living with overweight and obesity will benefit from body mass loss, strategies to attenuate the loss of FFM and muscle mass need to be developed.

There is some evidence reported that supplementation with long‐chain n‐3 polyunsaturated fatty acids (LCn‐3 PUFA), in the absence of other dietary or exercise interventions, can impact body composition by reducing body fat and increasing FFM [8, 9] and improve tight muscle volume, handgrip strength (HGS), and time to conduct the chair rising test (CRT) [10, 11]. The beneficial impact of LCn‐3 PUFA on body composition and muscle function can be attributed to several mechanisms, such as increasing muscle protein synthesis [12], enhancing mitochondrial content and function, and exerting anti‐inflammatory effects [13]. Some studies report that LCn‐3 PUFA during caloric restriction did not affect fat mass and FFM [14, 15], while others suggest that LCn‐3 PUFA during caloric restriction attenuated the reduction in FFM [16]. However, most of these studies utilized bioelectrical impedance analysis (BIA) techniques for measuring changes in FFM, which have multiple limitations [17]. On top of this, none of these studies assesses the effect of supplementation with LCn‐3 PUFA during diet‐induced weight loss on the parameters of muscle function. Furthermore, given that body weight loss [18] and EPA and DHA supplementation are both associated with reductions in inflammatory markers [19], it can be expected that LCn‐3 PUFA supplementation during body weight loss may positively influence muscle function by exerting a more pronounced anti‐inflammatory effect compared to body weight loss alone. Thus, further investigation is warranted.

The aim of the current study, therefore, is to investigate the effect of krill oil (KO) supplementation, as a source of LCn‐3 PUFA, on body composition, muscle function, and inflammatory markers during diet‐induced weight loss, via alternate‐day fasting, in adults living with overweight and obesity.

## 2 Methods

### 2.1 Participants

Healthy adults (25 women and 16 men) aged between 25 and 65 years with BMI > 25 kg/m^2^ were recruited through advertising posters and word of mouth in Glasgow City, UK. Inclusion criteria were nonsmokers, maintaining stable body weight for 1 month prior to study enrollment, and not taking any medication, nutritional supplements, or following a special diet. Female participants were also required to confirm that they were not pregnant. Exclusion criteria were chronic diseases, uncontrolled hypertension, allergies to seafood, or ambulatory impairments that would limit the ability to perform assessments of muscle function. Participants provided written informed consent after being provided with details on the study's objectives, risks, and potential discomfort. Ethical approval was obtained by the College of Medical, Veterinary, and Life Sciences Ethics Committee for Non‐Clinical Research Involving Human Participants [Reference 200210041].

### 2.2 Study Design

The current study was a double‐blind, randomized, placebo‐controlled trial with two parallel groups. Following the screening session, participants were randomly assigned to consume either 4 g/day of KO or vegetable oil (placebo) during the 8‐week alternate‐day fasting period. Before starting the 8‐week intervention, a 4‐week supplementation period was applied. Randomization was conducted in a 1:1 ratio, with the process carried out by an independent person using online software (https://www.sealedenvelope.com). To ensure that the study was blinded, an independent person labeled the capsules as either “A” or “B”, indicating the treatment or placebo group. This process ensured that neither the researchers nor the subjects knew whether they were receiving KO or a placebo. Participants conducted experimental trials after the 4‐week supplementation period (before) and after the 8‐week intervention involving the collection of biological samples, body weight measurements, saliva samples, and muscle function assessments. Additionally, participants recorded their food and drink intake for 3 days (including two weekdays and one weekend) before and during the final 3 days of the 8‐week intervention. Blood samples from an antecubital vein and dried blood spot (DBS) via finger stick were obtained before and after the intervention. The trial was registered at ClinicalTrials.gov (NCT06001632).

### 2.3 Intervention

#### 2.3.1 Alternate‐Day Fasting

The alternate‐day fasting regimen consists of a fasting day (24 h) followed by an ad libitum feeding day (24 h) [20], and this study included 28 fasting days and 28 feeding days. During fasting days, participants consumed only a prescribed meal of 500 kcal, comprising approximately 60 g of carbohydrates (CHO), 22 g of protein, 15 g of fat, and 10 g of fiber, which was to be consumed between 12 pm and 2 pm. For the fasting days, participants from both groups were provided with three meal suggestions that met the prescribed energy and macronutrient intake targets and were based on commonly available supermarket foods (Table S1). In addition to these meals, participants were advised to consume either bananas, blueberries, melons, or grapes in amounts providing no more than 50 kcal. They were allowed to consume water, coffee, tea, and noncaloric beverages, including diet sodas such as Coke Zero. During the feeding days, participants ate ad libitum and were advised to drink plenty of water. They were also advised on how to prevent overeating and, therefore, energy compensation following the fasting day. Participants were advised to avoid deviation from their habitual diet on feeding days. The participants were asked to start feeding and fasting days at midnight; however, actual adherence to the prescribed starting time was not monitored.

#### 2.3.2 Supplementation

Participants of the KO group were instructed to consume four capsules per day of KO, with each capsule containing 285 mg of LC n‐3 PUFA (191 mg of EPA, 94 mg of DHA), 78 mg of choline, and 100 mcg of astaxanthin. The placebo group was asked to take four capsules per day of mixed vegetable oil, comprising a blend of olive oil (extra virgin, cold‐pressed), maize oil (refined), palm kernel oil (refined), and medium‐chain triglycerides, in a ratio of 4:4:3:2, which was similar to the proportional distribution of fatty acids in a normal British diet. The supplements were provided as capsules, and participants took two capsules with lunch and two with dinner. They were contacted via emails, text messages, or phone calls weekly to maximize compliance with supplement intake, and compliance was assessed by comparing fatty acid composition in blood collected before and after the 8‐week intervention.

The KO and placebo capsules were provided free of charge by Aker BioMarine Human Ingredients AS (Lysker, Norway). The provider had no role in the study's design, conduct, or analysis.

### 2.4 Experimental Trials

The experimental trials conducted before and after the 8‐week intervention period were identical. Participants were asked to attend at ~9 am at the metabolic research unit at the New Lister Building of Glasgow Royal Infirmary, after a 12‐h fast. On arrival, the participants' body mass was measured. Following this, baseline saliva samples, using a passive cotton ball soaking sampling method, were collected from participants, followed by consumption of an accurately weighed dose of ~30 g of deuterium (D_2_O) (99.9%‐DLM4, Cambridge Isotope Laboratories Inc. MA, USA) for total body water (TBW) assessment. After dose consumption, ~50 mL of tap water was added to the tracer container and consumed to ensure complete ingestion. Then, a venous cannula was inserted to collect fasting blood samples. Follow‐up saliva samples were collected 3 h after ingestion of D_2_O. Then, the participants conducted the HGS test and CRT.

### 2.5 Anthropometric Measurements

Height was measured using a Harpenden wall‐mounted stadiometer (Holtain Ltd. Crymych, Pembs, UK). Body mass was measured using a balance scale (TANITA‐TBF‐310, UK). BMI was calculated as weight in kilograms divided by height in meters squared.

### 2.6 Total Body Water Measurements by Deuterium Dilution

TBW was measured from the saliva samples by D_2_O dilution using the stable isotope reference technique. D_2_O dilution is a well‐established method for the accurate determination of TBW, which is then used to calculate FFM and fat mass [21]. A two‐point plateau method was utilized to collect pre‐dose and post‐dose equilibration samples. The D_2_O dose was carefully prepared gravimetrically and diluted in local bottled water (~66% of the total solution).

The measurements of D_2_O enrichment (dilution space) in saliva samples were conducted using a portable Fourier Transform Infra‐Red (FTIR) spectrophotometer (Agilent 4500 t FTIR, Cheadle, UK). Alongside local QC standards, local water, and a diluted dose (approximating the dilution of body water), the baseline and post‐dose samples of each participant were measured in the same run, with water blanks between samples to monitor and reduce isotopic crossover between samples. Saliva ^2^H enrichment was calculated as post‐dose enrichment—baseline saliva ^2^H abundance. Standards and each saliva sample were measured in duplicate.

The ^2^H pool space (*V*_D_, kg) was calculated by saliva enrichment (mg/kg) ÷ dose (mg), and TBW was calculated from *V*_D_ correcting for the nonaqueous exchange factor (1.041) [22]. FFM (in kg) was calculated from TBW correcting for sex‐specific hydration factors, and fat mass (in kg) was calculated as the difference between body mass and FFM.

### 2.7 Handgrip Strength

HGS was measured three times in each hand, using a handheld hydraulic dynamometer (Jamar, Sammons Preston, Nottinghamshire, England, UK). Participants were seated with their arms supported and arms flexed to 90°. The highest value, in kg, was recorded and used as a value to be included in the analysis.

### 2.8 Chair Rising Test

The CRT has been used to evaluate leg power and knee extensor muscle size and strength. Participants were asked to sit on a chair with arms folded across their chest, perform a rise from a chair to a full standing position, and sit down again as quickly as possible five times, with time recorded for the completion of each attempt. The CRT was performed three times, and the fastest time was recorded and used as a value to be included in the analysis.

### 2.9 Blood Sampling and Analysis

Blood samples were collected from an antecubital vein into EDTA Vacuette tubes (Greiner Bio‐One, Kremsmünster, Austria) at pre‐ and post‐body mass loss intervention. The blood samples were centrifuged at 4°C, 3000 rpm for 15 min, and then the plasma was collected and frozen at −80°C for analysis. Plasma concentrations of TNF‐α, CRP, and IL‐6 were assessed using ELISA kits (enzyme‐linked immunosorbent assay) (Thermo Fisher Scientific Inc. UK). Plasma insulin concentration was assessed using an ELISA kit from Mercodia AB (Uppsala, Sweden). Plasma glucose and TAG concentrations were measured using enzymatic colorimetric kits (Randox Laboratories Ltd. Crumlin, UK; glucose: hexokinase method; TAG: GPO‐PAP method).

### 2.10 Fatty Acid Composition Analysis

A DBS was obtained at pre‐ and post‐intervention through a finger stick to analyze the proportional distribution of fatty acids in the blood. Specifically, a drop of blood was collected from participants and applied to a filter paper that had been pretreated with a cocktail solution (Fatty Acid Preservative Solution, FAPS; OmegaQuant LLC, USA). Afterward, the blood samples were then allowed to dry at room temperature for 15 min before being stored at −80°C until analysis. Samples were sent to the OmegaQuant laboratory, where the sample analyses were processed as previously reported (Harris & Jason Polreis). In brief, a single punch from the filter paper was carefully transferred into a glass vial with a screw cap followed by the addition of BTM methanol containing 14% boron trifluoride, toluene, methanol (35:30:35 v/v/v) (Sigma‐Aldrich, St. Louis, MO). The vial underwent a brief vortexing and was subjected to a 100°C hot bath for 45 min. After cooling, a mixture of hexane (from EMD Chemicals, USA) and HPLC‐grade water was added. The tubes were then recapped, vigorously vortexed, and centrifuged to facilitate the separation of layers. Following this separation, a sample from the hexane layer was carefully transferred to a gas chromatography (GC) vial. This extract was analyzed using a GC‐2030 Gas Chromatograph (Shimadzu Corporation, Columbia, MD) equipped with an SP‐2560 fused silica capillary column (100 m × 0.25 mm ID). The identification of the fatty acids was conducted by comparison with a standard mixture of fatty acids known to be characteristic of erythrocytes (GLC OQ‐A; NuCheck Prep, Elysian, MN, USA).

### 2.11 Dietary Intake Analysis

Energy and nutrient intake was assessed using a 3‐day weighted food diary method. Portable weighing scales (Salter Digital Kitchen Scale, Salter Housewares Ltd., UK) were provided to participants, and they were instructed to weigh and document all food and drink intake using the food diary form. The food diaries were then analyzed using Nutritix 2010 (Robert Gordon University, Aberdeen, Scotland, UK). A comparison of energy intake before the intervention and energy intake during the final 3 days of the intervention was used to test adherence to the alternate‐day fasting.

### 2.12 Statistical Analysis

Data normality was checked using the Shapiro–Wilk test. An independent *t*‐test was used to determine the differences between groups at baseline. A two‐way ANOVA with repeated measures was conducted to assess the impact of time, group, and time × group interaction on the study outcomes. All statistical analyses were conducted using IBM Statistical Package for the Social Sciences SPSS 28.0. Statistical significance was set at *p* < 0.05. Pearson's correlation coefficient was used to test associations between changes in FFM and changes in HGS and CRT.

The sample size was calculated based on HGS measurements, with a previously reported clinically significant difference ranging from 5.0 to 6.5 kg [23]. To detect a 6.5‐kg difference in HGS (standard deviation [SD] 7 kg based on our pilot data) with 80% power at a significance level of *p* < 0.05, 40 participants were required (20 participants per group). Accounting for potential dropouts, we recruited 52 participants.

## 3 Results

### 3.1 Participants

**FIGURE 1:**
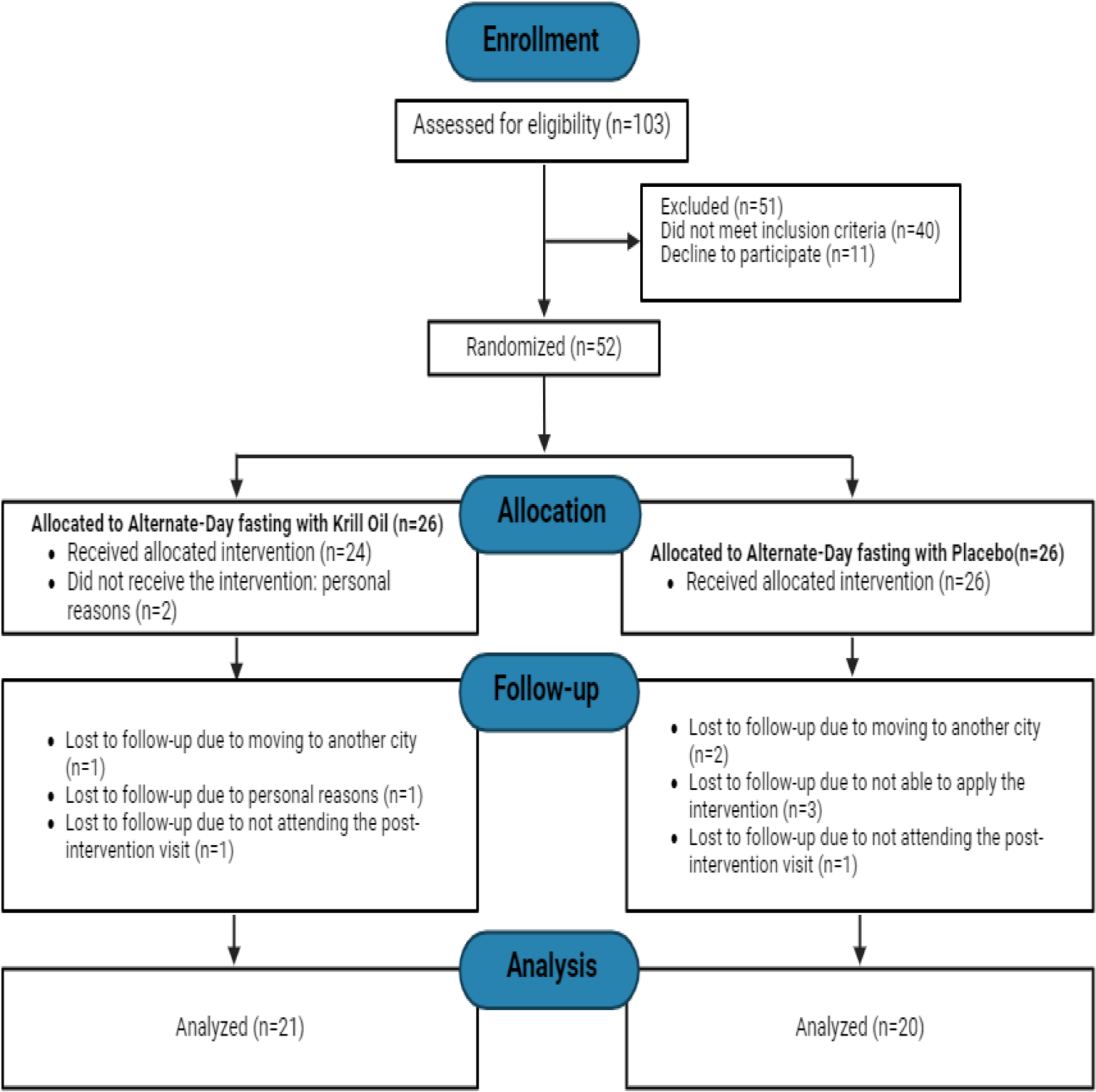
Flowchart diagram of the participants' selection and allocation. [Color figure can be viewed at wileyonlinelibrary.com]

Fifty‐two participants were enrolled in the study. After randomization, eleven participants withdrew from the study: three participants moved to another city, three were not able to follow the alternate‐day fasting diet, three withdrew for personal reasons, and two did not keep appointments for post‐intervention measurements. Therefore, 41 participants (25 women and 16 men) completed the study (Figure 1). At baseline, all measures did not differ (*p* > 0.05, independent *t*‐test) between groups (Table 1).

**TABLE 1:** Participants' characteristics, body mass, BMI, fat mass, FFM, handgrip strength, chair rising test, TNF‐α, CRP, IL‐6, and blood pressure measured before (pre) and after 8‐week intervention (post) and changes of these parameters in the KO (total, *n* = 21; men, *n* = 8; women, *n* = 13) and placebo (total, *n* = 20; men, *n* = 8; women, *n* = 12) groups.

	KO (*n* = 21)			Placebo (*n* = 20)			*p* values for time × group interaction
	Pre	Post	Δ	Pre	Post	Δ	
Age (y)	38.5 ± 10.8	*—*	***—***	40.2 ± 10	***—***	***—***	***—***
Height (cm)	165.5 ± 9	*—*	***—***	164.4 ± 8.12	***—***	***—***	***—***
Body mass (kg)	83.09 ± 14.2	78.4 ± 13.7***	−4.6 ± 1.4	85.3 ± 12.8	80.8 ± 12.8***	−4.5 ± 1.9	*p* > 0.05
BMI (kg/m^2^)	30.3 ± 3.9	28.6 ± 3.9***	−1.5 ± 0.6	31.8 ± 4.5	30.4 ± 4.5***	−1.4 ± 0.6	*p* > 0.05
FM (kg)	31.5 ± 6.1	27.1 ± 5.4***	−4.4 ± 1.7	32.4 ± 9	29.1 ± 7.9***	−3.3 ± 2.3	*p* > 0.05
FFM (kg)	51.5 ± 11.8	51.3 ± 11.7	−0.2 ± 0.9	52.9 ± 11.5	51. 7 ± 11*	−1.2 ± 2	***p* < 0.05**
HGS (kg)	35.7 ± 9.8	35.5 ± 9.9	−0.2 ± 0.5	32.2 ± 10	31.3 ± 9.8***	−0.9 ± 0.7	***p* < 0.05**
CRT (s)	9.1 ± 1.3	7.3 ± 1***	−1.8 ± 0.9	9.1 ± 1.6	8.8 ± 1.5	−0.3 ± 1.2	***p* < 0.05**
TNF‐α (pg/mL)	11.6 ± 2.4	10.2 ± 2.5***	−1.4 ± 0.2	13.2 ± 3.6	12.2 ± 3.5***	−0.9 ± 0.5	***p* < 0.05**
CRP (ng/mL)	1389 ± 206	1338 ± 201***	−51.4 ± 25	1466 ± 379	1432 ± 377***	−33.5 ± 12.6	***p* < 0.05**
IL‐6 (pg/mL)	2.2 ± 0.4	1.9 ± 0.4*	−0.3 ± 0.1	2.6 ± 0.3	2.4 ± 0.4*	−0.2 ± 0.1	*p* > 0.05
Systolic BP (mmHg)	122 ± 11	113 ± 12***	−9 ± 6	126 ± 7	122 ± 8***	−4 ± 4	***p* < 0.05**
Diastolic BP (mmHg)	73 ± 7	68 ± 8***	−5 ± 5	76 ± 8	73 ± 8***	−3 ± 3	*p* > 0.05
Insulin (mU/L)	8.4 ± 3.2	5.6 ± 1.8*	−2.8 ± 3.3	9.3 ± 2.8	8.1 ± 4	−1.2 ± 3.6	*p* > 0.05
Glucose (mmol/L)	5.9 ± 1.3	5.3 ± 1	−0.6 ± 0.9	6.02 ± 2	5.6 ± 0.8	−0.3 ± 1.3	*p* > 0.05
HOMA‐IR	2.2 ± 0.9	1.3 ± 0.5***	−0.9 ± 0.9	2.5 ± 1.2	2 ± 1.1	−0.5 ± 0.9	*p* > 0.05
TAG (mmol/L)	1.3 ± 0.5	1 ± 0.2*	−0.3 ± 0.5	1.1 ± 0.3	1.1 ± 0.3	−0.006 ± 0.4	*p* > 0.05

*Note*: Data presented as mean ± SD at different time points of intervention: pre, post, and Δ: change from baseline. At baseline, all measures were not different (*p* > 0.05, independent *t*‐test) between groups. Bold *p* values for time*group interaction indicate that changes between groups are significantly different.

Abbreviations: BP, blood pressure; CRP, C‐reactive protein; CRT, chair rising test; FFM, fat‐free mass; FM, fat mass; HGS, handgrip strength; IL‐6, interleukin‐6; TNF‐α, tumor necrosis factor‐alpha.

* Represents a significant difference from baseline within groups *p < 0.05; ***p < 0.001.

### 3.2 Body Weight and Body Composition

Body mass, BMI, fat mass, and FFM, measured before and after the 8‐week intervention, are presented in Table 1. Body weight was reduced by −4.6 ± 1.4 kg and −4.5 ± 1.9 kg in the KO and placebo groups, respectively. Two‐way ANOVA revealed significant time (*p* < 0.05) but not group (*p* > 0.05) or time × group interaction (*p* > 0.05) effects for body mass, BMI, and fat mass. For FFM, two‐way ANOVA showed significant time (*p* < 0.05) and time × group interaction effects (*p* < 0.0), but not a group (*p* > 0.05) effect, with FFM loss being significantly reduced in the placebo group (−1.2 ± 2.0 kg, *p* < 0.05) and not changed in the KO group (−0.2 ± 0.9 kg, *p* > 0.05).

### 3.3 Handgrip Strength and Chair Rising Test

HGS and time to complete the CRT, measured before and after the 8‐week intervention, are presented in Table 1. For HGS, two‐way ANOVA revealed significant time (*p* < 0.05) and time × group interaction (*p* < 0.05) effects, but not a group (*p* > 0.05) effect, with HGS being significantly reduced in the placebo group (−0.9 ± 0.7 kg, *p* < 0.05) but not changed in the KO group (−0.2 ± 0.5 kg, *p* > 0.05). For the time to complete the CRT, two‐way ANOVA revealed significant time (< 0.05) and time × group (*p* < 0.05) interaction effects, but not a group (*p* > 0.05) effect, with time to complete the CRT being shorter in the KO group (−1.8 ± 0.9 s, *p* < 0.05) but not changed in the placebo group (−0.3 ± 1.2 s, *p* > 0.05). In the pooled sample, changes in HGS were not correlated with FFM changes (*r* = 0.04, *p* = 0.82) while changes in the time to complete CRT were negatively correlated with changes in FFM (*r* = −0.37, *p* = 0.02).

### 3.4 Inflammatory Markers

Plasma concentrations of inflammatory markers measured before and after the 8‐week intervention are presented in Table 1. For TNF‐α and CRP plasma concentrations, two‐way ANOVA revealed significant time (*p* < 0.05) and time × group interaction (*p* < 0.05) effects, but not a group (*p* > 0.05) effect. In the KO group, the reduction in TNF‐α concentration (−1.4 ± 0.2 pg/mL) was significantly (*p* < 0.05) greater compared to the placebo group (−0.9 ± 0.5 pg/mL). Reduction in CRP concentration in the KO group (−51.4 ± 25 ng/mL) was also significantly (*p* < 0.05) greater than in the placebo group (−33.5 ± 12.6 ng/mL). Two‐way ANOVA revealed significant time (*p* < 0.05) but not group (*p* > 0.05) or time × group interaction (*p* > 0.05) effects for IL‐6 levels.

### 3.5 Insulin, Glucose, HOMA‐IR, TAG, and Blood Pressure

Plasma concentrations of insulin, glucose, HOMA‐IR, TAG, and blood pressure measured before and after the 8‐week intervention are presented in Table 1. For insulin and HOMA‐IR, two‐way ANOVA revealed significant time (*p* < 0.05), but not group (*p* > 0.05) or time × group interaction (*p* > 0.05). There was no time, group, or time × group interaction effect (all *p* > 0.05) for glucose.

For systolic blood pressure, two‐way ANOVA revealed significant time (*p* < 0.05), group (*p* < 0.05), and time × group interaction (*p* < 0.05) effects, with significantly (*p* < 0.05) greater reduction in the KO group (−9 ± 6 mmHg) than in the placebo group (−4 ± 4 mmHg). There was a significant time (*p* < 0.05) but no group (*p* > 0.05) or time × group interaction (*p* > 0.05) effect for diastolic blood pressure.

### 3.6 Energy Intake

Two‐way ANOVA revealed significant time (*p* < 0.05) but not group (*p* > 0.05) or time × group interaction (*p* > 0.05) effects for energy, CHO, protein, and fat intake measured before and during the last 3 days of the 8‐week intervention (Table 2).

**TABLE 2:** Energy, CHO, fat, protein, and n‐3 PUFA intake measured before (pre) and during the last 3 days of the 8‐week intervention (post) and changes in the intakes in the KO (total, *n* = 21; men, *n* = 8; women, *n* = 13) and placebo (total, *n* = 20; men, *n* = 8; women, *n* = 12) groups.

	KO (*n* = 21)			Placebo (*n* = 20)			*p* values for time × group interaction
	Pre	Post	Δ	Pre	Post	Δ	
Energy intake (kcal)	1833 ± 330	1269 ± 316 ***	−564 ± 330	1641 ± 331	1141 ± 236***	−500 ± 317	*p* > 0.05
CHO intake (g)	171 ± 49	122 ± 33***	−50 ± 41	164 ± 50	124 ± 26 ***	−39 ± 46	*p* > 0.05
Fat intake (g)	65 ± 18	42 ± 14***	−22 ± 16	64 ± 29	39 ± 12***	−24 ± 28	*p* > 0.05
Protein intake (g)	79 ± 24	62 ± 18*	−17 ± 19	80 ± 36	60 ± 25*	−19 ± 34	*p* > 0.05
Total n‐3 PUFA (g)	0.4 ± 0.6	0.3 ± 0.4	−0.1 ± 0.2	0.4 ± 0.3	0.2 ± 0.3	−0.2 ± 0.2	*p* > 0.05

*Note*: Data presented as mean ± SD at different time points of intervention: pre, post, and Δ: change from baseline.

Abbreviation: CHO, carbohydrate.

* Represents significant difference from baseline within groups *p < 0.05; ***p < 0.001. Time × group interaction: difference between groups.

### 3.7 Fatty Acid Composition

Two‐way ANOVA revealed significant time, group, and time × group interaction effects for the proportional distribution of EPA and DHA in blood and omega‐3 index (all *p* < 0.05), with these measures in the KO group relative to placebo being increased by 157% ± 109%, 58% ± 48%, and 55% ± 37%, respectively (Table 3).

**TABLE 3:** Proportional distribution (%) of fatty acids in blood at baseline and after 8‐week intervention (post) in the KO (total, *n* = 21; men, *n* = 8; women, *n* = 13) and placebo (total, *n* = 20; men, *n* = 8; women, *N* = 12) groups.

	KO (*n* = 21)		Placebo (*n* = 20)		*p* value for time × groupinteraction
	Baseline	Post	Baseline	Post	
Myristic acid	0.5 ± 0.2	0.6 ± 0.2	0.5 ± 0.2	0.5 ± 0.2	*p* > 0.05
Palmitic acid	21.1 ± 1.1	21.2 ± 1.2	21.7 ± 0.9	21.8 ± 1.1	*p* > 0.05
Palmitelaidic acid	0.2 ± 0.1	0.2 ± 0.2	0.2 ± 0.1	0.3 ± 0.2	*p* > 0.05
Palmitoleic acid	0.7 ± 0.3	0.8 ± 0.1	1.1 ± 0.5	1.1 ± 0.5	*p* > 0.05
Stearic acid	11.9 ± 2.3	11.8 ± 1.4	11.1 ± 0.9	11.1 ± 1.1	*p* > 0.05
Elaidic acid	0.3 ± 0.1	0.4 ± 0.2	0.3 ± 0.1	0.5 ± 0.4	*p* > 0.05
Oleic acid	18.6 ± 2.5	18.9 ± 3.4	20.2 ± 2.4	20.4 ± 2.3	*p* > 0.05
Linolelaidic acid	0.2 ± 0.1	0.3 ± 0.2	0.2 ± 0.2	0.4 ± 0.2	*p* > 0.05
Linoleic acid	20.8 ± 5.6	20.4 ± 3.8	20.2 ± 4.1	20.3 ± 3.6	*p* > 0.05
Arachidic acid	0.2 ± 0.05	0.2 ± 0.1	0.2 ± 0.1	0.2 ± 0.1	*p* > 0.05
Gamma‐linolenic acid	0.2 ± 0.1	0.2 ± 0.1	0.3 ± 0.1	0.3 ± 0.1	*p* > 0.05
Eicosenoic acid	0.2 ± 0.05	0.2 ± 0.1	0.2 ± 0.1	0.2 ± 0.1	*p* > 0.05
Alpha‐linolenic acid	0.4 ± 0.1	0.5 ± 0.1	0.4 ± 0.1	0.4 ± 0.1	*p* > 0.05
Eicosadienoic acid	0.2 ± 0.1	0.2 ± 0.1	0.2 ± 0.1	0.2 ± 0.1	*p* > 0.05
Behenic acid	0.8 ± 0.2	0.8 ± 0.3	0.9 ± 0.1	0.9 ± 0.1	*p* > 0.05
Dihomo‐g‐linolenic acid	1.3 ± 0.3	1.3 ± 0.2	1.5 ± 0.1	1.5 ± 0.1	*p* > 0.05
Arachidonic acid	11.1 ± 2.9	10.7 ± 1.9	10.2 ± 1.7	10.1 ± 1.3	*p* > 0.05
Lignoceric acid	1.3 ± 0.4	1.2 ± 0.2	1.4 ± 0.3	1.4 ± 0.2	*p* > 0.05
Eicosapentaenoic acid	0.7 ± 0.2	1.8 ± 0.3*	0.6 ± 0.5	0.5 ± 0.3	***p* < 0.05**
Nervonic acid	1.4 ± 0.3	1.4 ± 0.1	1.6 ± 0.4	1.5 ± 0.3	*p* > 0.05
Docosatetraenoic acid	1.6 ± 1.5	1.7 ± 1.5	1.3 ± 0.3	1.3 ± 0.3	*p* > 0.05
Docosapentaenoic acid—n6	0.5 ± 0.1	0.5 ± 0.1	0.4 ± 0.1	0.4 ± 0.1	*p* > 0.05
Docosapentaenoic acid—n3	1.4 ± 0.5	1.6 ± 0.7	1.2 ± 0.2	1.2 ± 0.2	*p* > 0.05
Docosahexaenoic acid	3.4 ± 0.8	4.6 ± 1*	2.9 ± 0.9	2.7 ± 0.9	***p* < 0.05**
Omega‐3 index	5.5 ± 0.9	8.2 ± 1.6*	5.4 ± 1.6	5.3 ± 1.4	***p* < 0.05**

*Note*: Data presented as mean ± SD. *Represents significant (*p* < 0.05) difference from baseline within groups. Bold *p* values for time*group interaction indicate that changes between groups are significantly different.

## 4 Discussion

This double‐blind, randomized, placebo‐controlled trial showed that daily supplementation with 1.14 g of LCn‐3 PUFA (EPA, 764 mg/day; DHA, 376 mg/day), in the form of KO, during diet‐induced weight loss, which achieved an average of a 4.5‐kg reduction in body weight via alternate‐day fasting, significantly attenuated the decline in FFM and HGS and reduced time to conduct the CRT in healthy adults living with overweight and obesity, with no effect on body mass or fat mass. Therefore, supplementation with KO could serve as a useful therapeutic strategy to attenuate the decline of FFM and muscle strength during weight loss.

Previous evidence reported that diet‐induced weight loss, without applying exercise, significantly reduces FFM [3, 24, 25] and muscle mass [26]. The current study has shown that KO supplementation during diet‐induced weight loss can help with the attenuation of FFM loss, with no effect on body mass or fat mass loss. This finding is consistent with evidence that supplementation with fish oil during a calorie‐restricted diet preserved FFM [16]. In contrast to our findings, there is evidence suggesting that LCn‐3 PUFA supplementation during diet‐induced weight loss did not affect FFM loss [14, 15, 27, 28, 29]. However, in these studies, the duration of the intervention was shorter (3–4 weeks) [14, 15, 27, 28] than in our study. Furthermore, our study used the D_2_O dilution method, which is an accurate method for assessing FFM in individuals living with obesity [17]. In addition, the previous studies applied either fish oil [27, 28, 29] or only EPA as a supplement [29], and only one of the studies used KO [15]. However, the daily provision of EPA and DHA in this KO study was lower (EPA, 151 mg/day; DHA, 65 mg/day) [15] than in our study (EPA, 764 mg/day; DHA, 376 mg/day). Thus, the benefits from supplementation with KO might be expected only when supplementation is longer than 4 weeks and the intake of LCn‐3 PUFA is reasonably high. When compared with fish oil studies, our findings support the notion that KO supplements, which contain over 55% of LCn‐3 PUFA in phospholipid form [30] and thus have enhanced bioavailability of LCn‐3 PUFA, potentially make KO more effective than fish oil [31]. Additionally, KO contains choline and astaxanthin, compounds not found in fish oil, that may play an important role in muscle function [32]. Choline is a precursor for acetylcholine, which is involved in neurotransmission and muscle contraction, and research has shown that choline supplementation can reduce muscle strength and mass gains during exercise training, with supplementation levels similar to those used in this study [33]. Astaxanthin, a carotenoid with antioxidant properties [34], has been shown to reduce muscle atrophy and fibrosis during immobilization in rats and may influence muscle function in humans [35], although the supplementation dose in these studies was approximately 10 times higher than that used in the current study. Further research, comparing the effectiveness of fish oil and KO, is required.

This study is the first to investigate the effect of supplementation with KO during diet‐induced weight loss on the parameters of muscle function. Previous evidence showed that supplementation with LCn‐3 PUFA, whether through KO or fish oil, increases muscle protein synthesis during a hyperinsulinaemic‐euglycemic clamp and improves HGS and physical performance [10, 11]. However, these studies [10, 11] investigated the impact of LCn‐3 PUFA supplements in the absence of caloric restriction. The current study, therefore, is the first to show that KO supplementation during diet‐induced weight loss attenuated the reduction in HGS and improved physical function, measured by the CRT. However, the observed effect of KO on grip strength in our study appears to be lower than the minimally clinically important difference (MCID) derived from our sample size calculation. This could be attributed to the relatively short duration of the intervention applied. Therefore, further research with longer durations and more advanced changes in body weight, FFM, and grip strength is needed.

The mechanisms behind the beneficial effects of LCn‐3 PUFA supplementation during diet‐induced weight loss on FFM and parameters of muscle function are not fully understood. Low‐grade inflammation is considered a contributor to a decline in lean body mass and strength [36], and it has been suggested that LCn‐3 PUFA have an anti‐inflammatory impact [37]. In the current study, KO supplementation during diet‐induced weight loss was associated with greater TNF‐α and CRP reduction, which is consistent with previous evidence related to CRP levels [38] and TNF‐α levels [39]. Therefore, the reduction of inflammation might be among the mechanisms by which LCn‐3 PUFA help preserve FFM and muscle function during weight loss. In addition, LCn‐3 PUFA enhance muscle protein synthesis, mitochondrial function, blood supply, and neuromuscular performance [12, 13].

The current study also reported that KO supplementation during diet‐induced weight loss due to alternate‐day fasting was associated with a greater reduction in systolic blood pressure compared to placebo. This finding is consistent with a study that observed greater reductions in systolic blood pressure among healthy adults living with obesity who followed a weight loss diet supplemented with EPA and DHA compared to those who followed a weight loss diet alone [40]. Similarly, daily fish consumption in men with hypertension resulted in greater reductions in both systolic and diastolic blood pressure during weight loss [41]. However, some studies using LCn‐3 PUFA doses of 1.2–3 g/day, which are close to or above the dose used in our study (1.14 g/day), reported no additional effects on blood pressure [42, 43]. A recent meta‐analysis suggests that blood pressure is lowered when the intake of LCn‐3 PUFA is between 2 g/day and 3 g/day [44], which is higher than the intake of LCn‐3 PUFA applied in our study (1.14 g/day).

Muscle function measurements were obtained only from HGS tests and CRT, which might be a limitation of this study. Another limitation is the relatively short duration of the intervention applied. In addition, the potential impact of the fasting‐induced reduction in body water [45] on FFM measurements obtained via the D_2_O dilution method represents a methodological limitation, as changes in hydration status may have influenced the accuracy of lean mass estimates. Therefore, our findings need to be confirmed by future studies employing more precise techniques of body composition measurements such as neutron activation analysis [46], whole‐body magnetic resonance imaging, and extracellular water (ECW) by bromide dilution [47]. Additionally, the lack of physical activity assessment might be considered another limitation. On the other hand, study participants were sedentary at the point of recruitment and reported no prior experience with any planned and structured exercise, including resistance training. Thus, we did not expect them to engage in exercise training during the intervention period. Furthermore, the suboptimal protein intake of our participants during the diet‐induced weight loss period may have contributed to the loss of FFM observed [48]. This, however, reflects real‐world adherence to the alternate‐day fasting diet and enhances the external validity of our study, and as such, we consider this a strength. Therefore, it would be of clear interest in future research to investigate whether the beneficial effects of KO on FFM and muscle function persist when protein intake is optimal during alternate‐day fasting.

A further limitation is the use of DBS samples for fatty acid analysis, as this method reflects a mixture of plasma and erythrocyte fatty acid profiles and may not capture long‐term dietary intake as accurately as red blood cell analysis alone [49]. We appreciate that the lack of a comparison of the outcomes between males and females is another limitation of our study. At the same time, sex‐specific analyses were not within the scope of this study. We could not identify the justification for a diverse response between males and females for the main outcomes of this study, and power calculations for variables of interest were conducted for the entire sample. Finally, dietary intake before and during the last week of the intervention was assessed using 3‐day rather than 7‐day weighted food diaries. This may have reduced the accuracy of true energy and macronutrient intake estimation.

On the other hand, the current study has some strengths. This was the first study to investigate the effect of KO during diet‐induced weight loss on the parameters of muscle function. Additionally, in the current study, fat mass and FFM assessments were conducted using the D_2_O method, incorporating the quantification of TBW volumes, which provides a more accurate and reliable measurement of body composition compared to alternative methods like BIA and DXA [17]. Furthermore, participants showed strong adherence to the supplements, as evidenced by a significant increase in EPA, DHA, and omega‐3 index in the blood at post‐intervention in the KO group. Additionally, compliance with the diet was observed, as participants achieved predicted weight loss, and energy intake was significantly reduced in both groups at post‐intervention.

## 5 Conclusion

Supplementation with KO during body weight loss in individuals living with overweight and obesity attenuates the decline in FFM and parameters of muscle function and, therefore, is a valuable strategy to mitigate some of the adverse effects of diet‐induced weight loss. The anti‐inflammatory effects of KO might be among the mechanisms underlying these benefits.

## Author Contributions

All authors contributed to the conception and development of the study. Mansour Alblaji, Dalia Malkova, and Stuart Gray designed the study. Mansour Alblaji, Dalia Malkova, and Taibah Almesbehi conducted the study and analyzed the data. Douglas Morrison oversaw the isotope work. Mansour Alblaji and Dalia Malkova wrote the first draft of the manuscript, and all authors conducted multiple rounds of critical review and revision. All authors agree to be fully accountable for ensuring the integrity and accuracy of the work, and all authors affirm that they have read and approved the final manuscript.

## Conflicts of Interest

The authors declare no conflicts of interest.

## Supporting information


**Data S1.**oby24354‐sup‐0001‐supinfo.

## Data Availability

The data that support the findings of this study are available on request from the corresponding author.

## References

[1] T. Kelly , W. Yang , C.‐S. Chen , K. Reynolds , and J. He , “Global Burden of Obesity in 2005 and Projections to 2030,” International Journal of Obesity 32 (2008): 1431–1437. 18607383 10.1038/ijo.2008.102

[2] L. Ge , B. Sadeghirad , G. D. Ball , et al., “Comparison of Dietary Macronutrient Patterns of 14 Popular Named Dietary Programmes for Weight and Cardiovascular Risk Factor Reduction in Adults: Systematic Review and Network Meta‐Analysis of Randomised Trials,” BMJ 369 (2020): m696. 32238384 10.1136/bmj.m696 PMC7190064

[3] J. Turicchi , R. O'Driscoll , G. Finlayson , et al., “Associations Between the Proportion of Fat‐Free Mass Loss During Weight Loss, Changes in Appetite, and Subsequent Weight Change: Results From a Randomized 2‐Stage Dietary Intervention Trial,” American Journal of Clinical Nutrition 111 (2020): 536–544. 31950141 10.1093/ajcn/nqz331

[4] T. B. Chaston , J. Dixon , and P. E. O'Brien , “Changes in Fat‐Free Mass During Significant Weight Loss: A Systematic Review,” International Journal of Obesity 31 (2007): 743–750. 17075583 10.1038/sj.ijo.0803483

[5] J. Zibellini , R. Seimon , C. Lee , A. Gibson , M. Hsu , and A. Sainsbury , “Effect of Diet‐Induced Weight Loss on Muscle Strength in Adults With Overweight or Obesity–a Systematic Review and Meta‐Analysis of Clinical Trials,” Obesity Reviews 17 (2016): 647–663. 27126087 10.1111/obr.12422

[6] A. Bosy‐Westphal , B. Schautz , M. Lagerpusch , et al., “Effect of Weight Loss and Regain on Adipose Tissue Distribution, Composition of Lean Mass and Resting Energy Expenditure in Young Overweight and Obese Adults,” International Journal of Obesity 37 (2013): 1371–1377. 23381557 10.1038/ijo.2013.1

[7] J. C. Lagacé , M. Brochu , and I. J. Dionne , “A Counterintuitive Perspective for the Role of Fat‐Free Mass in Metabolic Health,” Journal of Cachexia, Sarcopenia and Muscle 11 (2020): 343–347. 31999082 10.1002/jcsm.12520 PMC7113531

[8] E. E. Noreen , M. J. Sass , M. L. Crowe , V. A. Pabon , J. Brandauer , and L. K. Averill , “Effects of Supplemental Fish Oil on Resting Metabolic Rate, Body Composition, and Salivary Cortisol in Healthy Adults,” Journal of the International Society of Sports Nutrition 7 (2010): 31. 20932294 10.1186/1550-2783-7-31 PMC2958879

[9] D. M. Crestani , É. F. R. Bonin , R. A. Barbieri , A. M. Zagatto , W. P. Higino , and F. Milioni , “Chronic Supplementation of Omega‐3 Can Improve Body Composition and Maximal Strength, but Does Not Change the Resistance to Neuromuscular Fatigue,” Sport Sciences for Health 13 (2017): 259–265.

[10] G. I. Smith , S. Julliand , D. N. Reeds , D. R. Sinacore , S. Klein , and B. Mittendorfer , “Fish Oil–Derived *n*− 3 PUFA Therapy Increases Muscle Mass and Function in Healthy Older Adults,” American Journal of Clinical Nutrition 102 (2015): 115–122. 25994567 10.3945/ajcn.114.105833 PMC4480667

[11] S. A. Alkhedhairi , F. F. A. Alkhayl , A. D. Ismail , et al., “The Effect of Krill Oil Supplementation on Skeletal Muscle Function and Size in Older Adults: A Randomised Controlled Trial,” Clinical Nutrition 41 (2022): 1228–1235. 35504165 10.1016/j.clnu.2022.04.007

[12] G. I. Smith , P. Atherton , D. N. Reeds , et al., “Dietary Omega‐3 Fatty Acid Supplementation Increases the Rate of Muscle Protein Synthesis in Older Adults: A Randomized Controlled Trial,” American Journal of Clinical Nutrition 93 (2011): 402–412. 21159787 10.3945/ajcn.110.005611 PMC3021432

[13] S. R. Gray and B. Mittendorfer , “Fish Oil‐Derived n‐3 Polyunsaturated Fatty Acids for the Prevention and Treatment of Sarcopenia,” Current Opinion in Clinical Nutrition and Metabolic Care 21 (2018): 104–109. 29232264 10.1097/MCO.0000000000000441

[14] I. A. Munro and M. L. Garg , “Dietary Supplementation With n‐3 PUFA Does Not Promote Weight Loss When Combined With a Very‐Low‐Energy Diet,” British Journal of Nutrition 108 (2012): 1466–1474. 22214842 10.1017/S0007114511006817

[15] A. Paoli , T. Moro , G. Bosco , et al., “Effects of n‐3 Polyunsaturated Fatty Acids (ω‐3) Supplementation on Some Cardiovascular Risk Factors With a Ketogenic Mediterranean Diet,” Marine Drugs 13 (2015): 996–1009. 25689563 10.3390/md13020996 PMC4344614

[16] S. Y. Huang , N. Sabrina , Y. W. Chien , Y. C. Chen , S. H. Lin , and J. S. Chang , “A Moderate Interleukin‐6 Reduction, Not a Moderate Weight Reduction, Improves the Serum Iron Status in Diet‐Induced Weight Loss With Fish Oil Supplementation,” Molecular Nutrition & Food Research 62 (2018): 1800243. 10.1002/mnfr.201800243 30052315

[17] B. Jensen , W. Braun , C. Geisler , et al., “Limitations of Fat‐Free Mass for the Assessment of Muscle Mass in Obesity,” Obesity Facts 12 (2019): 307–315. 31132777 10.1159/000499607 PMC6696776

[18] A. B. Aamir , R. Kumari , R. Latif , et al., “Effects of Intermittent Fasting and Caloric Restriction on Inflammatory Biomarkers in Individuals With Obesity/Overweight: A Systematic Review and Meta‐Analysis of Randomized Controlled Trials,” Obesity Reviews 26 (2024): e13838. 39289905 10.1111/obr.13838

[19] Z. Kavyani , V. Musazadeh , S. Fathi , A. H. Faghfouri , P. Dehghan , and B. Sarmadi , “Efficacy of the Omega‐3 Fatty Acids Supplementation on Inflammatory Biomarkers: An Umbrella Meta‐Analysis,” International Immunopharmacology 111 (2022): 109104. 35914448 10.1016/j.intimp.2022.109104

[20] K. A. Varady , S. Bhutani , M. C. Klempel , et al., “Alternate Day Fasting for Weight Loss in Normal Weight and Overweight Subjects: A Randomized Controlled Trial,” Nutrition Journal 12 (2013): 146, 10.1186/1475-2891-12-146. 24215592 PMC3833266

[21] M. Valencia , H. Alemán‐Mateo , G. Salazar , and M. Hernandez Triana , “Body Composition by Hydrometry (Deuterium Oxide Dilution) and Bioelectrical Impedance in Subjects Aged >60 y From Rural Regions of Cuba, Chile and Mexico,” International Journal of Obesity 27 (2003): 848–855. 12821972 10.1038/sj.ijo.0802315

[22] International Atomic Energy Agency , Introduction to Body Composition Assessment Using the Deuterium Dilution Technique With Analysis of Saliva Samples by Fourier Transform Infrared Spectrometry (IAEA, 2011).

[23] R. W. Bohannon , “Minimal Clinically Important Difference for Grip Strength: A Systematic Review,” Journal of Physical Therapy Science 31 (2019): 75–78. 30774209 10.1589/jpts.31.75 PMC6348186

[24] E. P. Weiss , R. C. Jordan , E. M. Frese , S. G. Albert , and D. T. Villareal , “Effects of Weight Loss on Lean Mass, Strength, Bone, and Aerobic Capacity,” Medicine and Science in Sports and Exercise 49 (2017): 206–217. 27580151 10.1249/MSS.0000000000001074 PMC5161655

[25] P. Chomentowski , J. J. Dubé , F. Amati , et al., “Moderate Exercise Attenuates the Loss of Skeletal Muscle Mass That Occurs With Intentional Caloric Restriction–Induced Weight Loss in Older, Overweight to Obese Adults,” Journals of Gerontology. Series A, Biological Sciences and Medical Sciences 64 (2009): 575–580. 19276190 10.1093/gerona/glp007 PMC2800807

[26] T. N. Frimel , D. R. Sinacore , and D. T. Villareal , “Exercise Attenuates the Weight‐Loss‐Induced Reduction in Muscle Mass in Frail Obese Older Adults,” Medicine and Science in Sports and Exercise 40 (2008): 1213–1219. 18580399 10.1249/MSS.0b013e31816a85ce PMC2650077

[27] C. Couet , J. Delarue , P. Ritz , J. Antoine , and F. Lamisse , “Effect of Dietary Fish Oil on Body Fat Mass and Basal Fat Oxidation in Healthy Adults,” International Journal of Obesity 21 (1997): 637–643. 15481762 10.1038/sj.ijo.0800451

[28] I. A. Munro and M. L. Garg , “Prior Supplementation With Long Chain Omega‐3 Polyunsaturated Fatty Acids Promotes Weight Loss in Obese Adults: A Double‐Blinded Randomised Controlled Trial,” Food & Function 4 (2013): 650–658. 23396496 10.1039/c3fo60038f

[29] A. E. Huerta , S. Navas‐Carretero , P. L. Prieto‐Hontoria , J. A. Martínez , and M. J. Moreno‐Aliaga , “Effects of α‐Lipoic Acid and Eicosapentaenoic Acid in Overweight and Obese Women During Weight Loss,” Obesity 23 (2015): 313–321. 25594166 10.1002/oby.20966

[30] J. C. Tou , J. Jaczynski , and Y.‐C. Chen , “Krill for Human Consumption: Nutritional Value and Potential Health Benefits,” Nutrition Reviews 65 (2007): 63–77. 17345959 10.1111/j.1753-4887.2007.tb00283.x

[31] S. M. Ulven and K. B. Holven , “Comparison of Bioavailability of Krill Oil Versus Fish Oil and Health Effect,” Vascular Health and Risk Management 11 (2015): 511–524. 26357480 10.2147/VHRM.S85165 PMC4559234

[32] S. Z. Liu , A. S. Ali , M. D. Campbell , et al., “Building Strength, Endurance, and Mobility Using an Astaxanthin Formulation With Functional Training in Elderly,” Journal of Cachexia, Sarcopenia and Muscle 9 (2018): 826–833. 30259703 10.1002/jcsm.12318 PMC6204600

[33] C. W. Lee , E. Galvan , T. Lee , et al., “Low Intake of Choline Is Associated With Diminished Strength and Lean Mass Gains in Older Adults,” Journal of Frailty & Aging 12 (2023): 78–83. 36629089 10.14283/jfa.2022.50

[34] R. Janani , R. E. Anitha , M. K. Perumal , P. Divya , and V. Baskaran , “Astaxanthin Mediated Regulation of VEGF Through HIF1α and XBP1 Signaling Pathway: An Insight From ARPE‐19 Cell and Streptozotocin Mediated Diabetic Rat Model,” Experimental Eye Research 206 (2021): 108555. 33789142 10.1016/j.exer.2021.108555

[35] T. Shibaguchi , Y. Yamaguchi , N. Miyaji , et al., “Astaxanthin Intake Attenuates Muscle Atrophy Caused by Immobilization in Rats,” Physiological Reports 4 (2016): e12885. 27482075 10.14814/phy2.12885 PMC4985550

[36] S. Dalle , L. Rossmeislova , and K. Koppo , “The Role of Inflammation in Age‐Related Sarcopenia,” Frontiers in Physiology 8 (2017): 1045. 29311975 10.3389/fphys.2017.01045 PMC5733049

[37] P. C. Calder , “Omega‐3 Fatty Acids and Inflammatory Processes,” Nutrients 2 (2010): 355–374. 22254027 10.3390/nu2030355 PMC3257651

[38] F. A. Utami , H.‐C. Lee , C.‐T. Su , Y.‐R. Guo , Y.‐T. Tung , and S.‐Y. Huang , “Effects of Calorie Restriction Plus Fish Oil Supplementation on Abnormal Metabolic Characteristics and the Iron Status of Middle‐Aged Obese Women,” Food & Function 9 (2018): 1152–1162. 29362766 10.1039/c7fo01787a

[39] I. A. Munro and M. L. Garg , “Dietary Supplementation With Long Chain Omega‐3 Polyunsaturated Fatty Acids and Weight Loss in Obese Adults,” Obesity Research & Clinical Practice 7 (2013): 173–181. 10.1016/j.orcp.2011.11.001 23697585

[40] A. T. Wong , D. C. Chan , P. H. R. Barrett , L. A. Adams , and G. F. Watts , “Effect of ω‐3 Fatty Acid Ethyl Esters on Apolipoprotein B‐48 Kinetics in Obese Subjects on a Weight‐Loss Diet: A New Tracer Kinetic Study in the Postprandial State,” Journal of Clinical Endocrinology and Metabolism 99 (2014): E1427–E1435. 24606094 10.1210/jc.2013-4037

[41] D. Q. Bao , T. A. Mori , V. Burke , I. B. Puddey , and L. J. Beilin , “Effects of Dietary Fish and Weight Reduction on Ambulatory Blood Pressure in Overweight Hypertensives,” Hypertension 32 (1998): 710–717. 9774368 10.1161/01.hyp.32.4.710

[42] L. F. DeFina , L. G. Marcoux , S. M. Devers , J. P. Cleaver , and B. L. Willis , “Effects of Omega‐3 Supplementation in Combination With Diet and Exercise on Weight Loss and Body Composition,” American Journal of Clinical Nutrition 93 (2011): 455–462. 21159785 10.3945/ajcn.110.002741

[43] H.‐C. Lee , W.‐Y. Cheng , Y.‐H. Hsu , et al., “Effects of Calorie Restriction With n‐3 Long‐Chain Polyunsaturated Fatty Acids on Metabolic Syndrome Severity in Obese Subjects: A Randomize‐Controlled Trial,” Journal of Functional Foods 19 (2015): 929–940.

[44] X. Zhang , J. A. Ritonja , N. Zhou , B. E. Chen , and X. Li , “Omega‐3 Polyunsaturated Fatty Acids Intake and Blood Pressure: A Dose‐Response Meta‐Analysis of Randomized Controlled Trials,” Journal of the American Heart Association 11 (2022): e025071. 35647665 10.1161/JAHA.121.025071 PMC9238708

[45] M. T. Najafi , A. Sadoogh Abbasian , H. Mohammadi , et al., “Alteration in Body Water Compartments Following Intermittent Fasting in Ramadan,” Frontiers in Nutrition 10 (2023): 1232979. 37645631 10.3389/fnut.2023.1232979 PMC10461004

[46] J. J. Kehayias and S. Valtueña , “Neutron Activation Analysis Determination of Body Composition,” Current Opinion in Clinical Nutrition and Metabolic Care 2 (1999): 453–463. 10678673 10.1097/00075197-199911000-00004

[47] D. G. Levitt , L. M. Beckman , J. R. Mager , et al., “Comparison of DXA and Water Measurements of Body Fat Following Gastric Bypass Surgery and a Physiological Model of Body Water, Fat, and Muscle Composition,” Journal of Applied Physiology 109 (2010): 786–795. 20558754 10.1152/japplphysiol.00278.2010 PMC2944634

[48] H. J. Leidy , N. S. Carnell , R. D. Mattes , and W. W. Campbell , “Higher Protein Intake Preserves Lean Mass and Satiety With Weight Loss in Pre‐Obese and Obese Women,” Obesity 15 (2007): 421–429. 17299116 10.1038/oby.2007.531

[49] W. S. Harris and J. Polreis , “Measurement of the Omega‐3 Index in Dried Blood Spots,” Annals of Clinical and Laboratory Research 4 (2016): 137.

