# Supplementary material for: Benefits of Krill Oil Supplementation During Alternate‐Day Fasting in Adults With Overweight and Obesity: A Randomized Trial
Source: Obesity (Silver Spring). 2025 Jul 16;33(9):1694–703. doi: 10.1002/oby.24354 (PMC12381611; doi:10.1002/oby.24354)
Supplement: Supplementary file 1 — Data S1.oby24354‐sup‐0001‐supinfo. [file OBY-33-1694-s001.docx]

**Supplementary Materials**

**Benefits of krill oil supplementation during alternate-day fasting in adults with overweight and obesity: A randomized trial**

Mansour Alblaji [**^1^**](#affiliation1)**^,^** [**^4^**](#affiliation4), Stuart R. Gray [**^2^**](#affiliation2)**^,^** [**^5^**](#affiliation5), Taibah Almesbehi [**^1^**](#affiliation1), Douglas J. Morrison [**^3^**](#affiliation3), & Dalia Malkova [**^1^**](#affiliation1)

**^1^** Human Nutrition, School of Medicine, Dentistry and Nursing, College of Medical, Veterinary, and Life Sciences, University of Glasgow, New Lister Building, Glasgow Royal Infirmary, Glasgow, G31 2ER, UK.

**^2^** School of Cardiovascular and Metabolic Health, College of Medical, Veterinary and Life Sciences, University of Glasgow, Glasgow, G12 8TA, UK

**^3^** Scottish Universities Environmental Research Centre (SUERC), University of Glasgow, East Kilbride, G75 0QF, UK.

**^4^** Department of Basic Health Sciences, College of Applied Medical Sciences, Qassim University, Buraydah 52571, Saudi Arabia.

**^5^** Institute of Sports Science and Innovation; Lithuanian Sports University, Sporto g, Kaunas 44221, Lithuania.

**TABLE** [**S1**](#TABLES1) The energy and nutrients of the meals suggested for the fasting days

|  | **Meal 1** | **Meal 2** | **Meal 3** |
| --- | --- | --- | --- |
|  | Chicken tomato and Basal Pasta (300 g) | Vegetarian pizza (210 g) | Tomato and Mozzarella pasta (400g) |
| **Energy (kcal)** | 460 | 390 | 419 |
| **Total fat (g)** | 16 | 10 | 11 |
| **Saturated fat (g)** | 1.5 | 3.8 | 4.6 |
| **Total Carbohydrate (g)** | 61 | 57 | 61 |
| **Sugars (g)** | 12 | 9 | 14 |
| **Protein (g)** | 18 | 18 | 19 |
| **Fibre (g)** | 4 | 5 | 6 |
| **Salt** | 1.2 | 1.6 | 1.4 |

***Note****:* In addition to these meals, participants were advised to consume either bananas, blueberries, melon or grapes in amounts providing no more than 50 kcal.
